# Icariin Regulates Cellular Functions and Gene Expression of Osteoarthritis Patient-Derived Human Fibroblast-Like Synoviocytes

**DOI:** 10.3390/ijms18122656

**Published:** 2017-12-08

**Authors:** Lianhong Pan, Yonghui Zhang, Na Chen, Li Yang

**Affiliations:** 1 Department of Basic Medicine, Chongqing Three Gorges Medical College, Chongqing 404000, China; donglilibme@cqu.edu.cn (L.P.); weiyong@hrbeu.edu.cn (Y.Z.); 2 Digital Medicine Institute, Biomedical Engineering College, Third Military Medical University, Chongqing 400038, China; 3 National Innovation and Attracting Talents “111” Base, Key Laboratory of Biorheological Science and Technology, Ministry of Education, College of Bioengineering, Chongqing University, Chongqing 400030, China; yanglibme@cqu.edu.cn

**Keywords:** icariin, osteoarthritis, OA–FLSs, anti-inflammation, *MMP14*, *GRP78*

## Abstract

Synovial inflammation plays an important role in the pathogenesis and progress of osteoarthritis (OA). There is an urgent need to find safe and effective drugs that can reduce the inflammation and regulate the pathogenesis of cytokines of the OA disease. Here, we investigated the effect of icariin, the major pharmacological active component of herb *Epimedium* on human osteoarthritis fibroblast-like synoviocytes (OA–FLSs). The OA–FLSs were isolated from patients with osteoarthritis and cultured in vitro with different concentrations of icariin. Then, cell viability, proliferation, and migration were investigated; *MMP14*, *GRP78*, and *IL-1β* gene expression levels were detected via qRT-PCR. Icariin showed low cytotoxicity to OA–FLSs at a concentration of under 10 μM and decreased the proliferation of the cells at concentrations of 1 and 10 μM. Icariin inhibited cell migration with concentrations ranging from 0.1 to 1 μM. Also, the expression of three cytokines for the pathogenesis of OA which include *IL-1β*, *MMP14* and *GRP78* was decreased by the various concentrations of icariin. These preliminary results imply that icariin might be an effective compound for the treatment of OA disease.

## 1. Introduction

Osteoarthritis (OA) is a chronic joint bone disease characterized by the progressive damage of joint structure including articular cartilage and subchondral bone which always leads to the dysfunction and disability of arthritis [1]. Causes of the OA disease mainly include age, obesity, joint damage and re-injury as well as the early onset of diabetes [2]. However, a growing body of studies suggest that the activated fibroblast-like synoviocytes (FLSs) play an important role in OA pathogenesis [3,4]. Condensed cells form the normal synovium—mainly FLSs—of which the lining layer is 1–3 cells thick [5]. FLSs provide nutrients for articular cartilage and protect the joint structures and the adjacent musculoskeletal tissues.

Though OA is always regarded as a non-inflammatory joint disease, it is widely accepted that synovitis has effects on both symptoms and progression of OA. The synovial inflammation is frequently involved in the process of OA, even at an early stage [6,7]. In addition, inflamed OA synovial tissue might release inflammatory mediators such as interleukin (IL)-6, tumor necrosis factor (TNF)-α and matrix metalloproteinases (MMPs). In the progression of OA, the changes of synovial histology include synovial hypertrophy and hyperplasia with large amounts of lining cells usually accompanied by lymphocyte infiltration [8]. Previous research data indicate that FLSs play an important role in OA cartilage degradation by producing inflammatory and catabolic mediators [9,10,11]. It has been demonstrated that the proliferation of OA–FLSs could have an effect on both the morphology and proliferation of cartilage cells [12], and the proliferation and migration of OA–FLSs increase the number of cells with many inflammatory cytokines which may lead to hypertrophy and hyperplasia. Hypertrophy is one of the typical pathological processes of the OA disease.

Some studies report that FLSs can secrete large amounts of inflammatory cytokines such as *IL-1* in the stimulation conditions [13,14]. Therefore, FLSs are important to regulate the inflammation of synovium which can result in cartilage degradation and disease pathology [15]. Moreover, the degradation of the extracellular matrix (ECM) is another key factor of the occurrence of OA. Studies reported that FLSs produce enzymes to degrade the ECM, such as MMPs which have been found to be over-activated in OA [16], including *MMP14*, which is one of the membrane-type MMPs and has a critical effect on enhancing cell invasion and migration in tumor cells [17]. In recent years, studies have shown that *MMP14* functions in the cartilage tissue in OA disease by degrading the collage II directly and activating other members of MMPs such as *MMP13* [18]. However, due to the fact that FLSs are the main cells in the articular cavity which could establish a three-dimensional complex synovial lining architecture and produce synovial fluid constituents [19], they play an important role in regulating the progress of OA. The accumulation of the apoptosis FLSs may aggravate the microenvironment of the joint cavity by leading to many inflammation cytokines; therefore, the apoptosis of FLSs would accelerate the OA process. Glucose-regulated protein-78 (*GRP78*) is a major endoplasmic reticulum (ER) chaperone and widely used as a marker for ER stress by regulating the unfold protein response (UPR) signaling pathway [20]. Although evidence of *GRP78* expression with OA–FLS apoptosis is lacking, it has been proven that excessive ER stress can lead to cell death mediated by apoptosis [21,22]. The high expression of *GRP78* could promote the apoptosis of the FLSs which could regulate the composition and homeostasis of synovium, further affect the pathogenesis of OA and then lead to synovial inflammation and hyperplasia. Therefore, regulating the function of FLSs and inhibiting the apoptosis of FLSs might be seen as a good way to treat OA disease.

The Traditional Chinese Medicine theory shows that the traditional Chinese herbal medicine herb *Epimedium* (HEP) has therapeutic potential for the treatment of OA. Icariin (C_33_H_40_O_15_; molecular weight: 676.67), is a natural product isolated from HEP that is proven to have antioxidative [23] and anti-inflammatory properties [24]. Further studies demonstrated that icariin can inhibit osteoclast differentiation, prevent inflammatory bone loss [25], and protect chondrocytes from inflammation in septic arthritis [26]. However, to date, there is no report on the influence of icariin on the main cells in synovium—FLSs. Therefore, we hypothesized that icariin could have an effect on OA–FLSs in three aspects: inhibiting the proliferation of the cells; preventing the migration of cells; and regulating cells by reducing the inflammation, degradation of ECM, and preventing the apoptosis of the cells. In the present work, we found that icariin could inhibit the cell migration and proliferation, thus preventing the expression of cytokine *IL-1β* which further reduces the inflammatory response. Also, it can reduce the *MMP14* expression level as well as inhibit the ER stress. These results indicate that icariin might be a promising compound for the treatment of OA disease.

## 2. Results

### 2.1. Effect of Icariin on the Osteoarthritis Fibroblast-Like Synoviocytes (OA–FLSs) Viability, Proliferation and Migration

**Figure 1:**
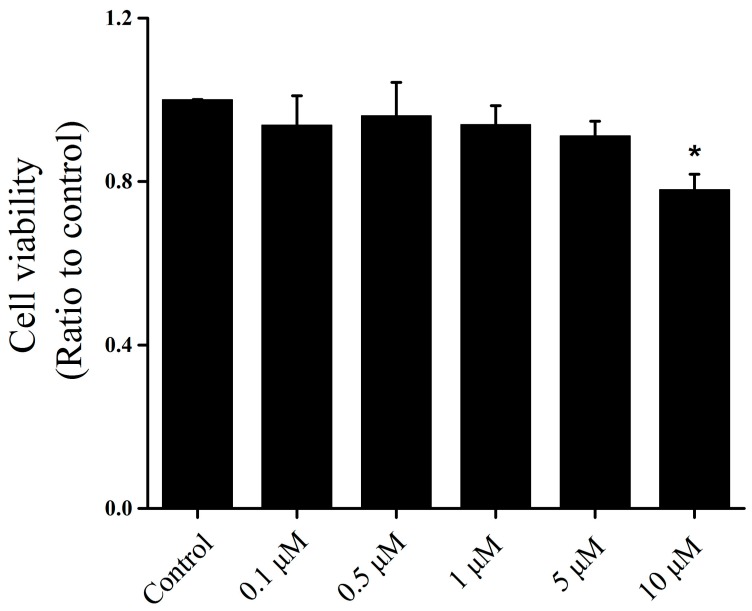
Effects of icariin on cell viability of osteoarthritis fibroblast-like synoviocytes (OA–FLSs). Cells were incubated with different concentrations of icariin for 12 h; cell metabolic activity and proliferation were measured by MTS assay. * *p* < 0.05 were accepted as statistically significant (mean ± SD, *n* = 4).

In our preliminary experiments, we detected cytotoxic effects of icariin on OA–FLSs at different doses by 3-(4,5-dimethylthiazol-2-yl)-5-(3-carboxymethoxyphenyl)-2-(4-sulfophenyl)-2H-tetrazolium (MTS) assay (Figure 1). The results show that no cytotoxic effects of icariin at 0.1, 0.5, 1 μM concentration were observed in the OA–FLSs. However, the icariin showed low cytotoxicity to OA–FLSs at a concentration of under 10 μM of icariin which significantly suppressed the proliferation of OA–FLSs after 12 h (Figure 1).

**Figure 2:**
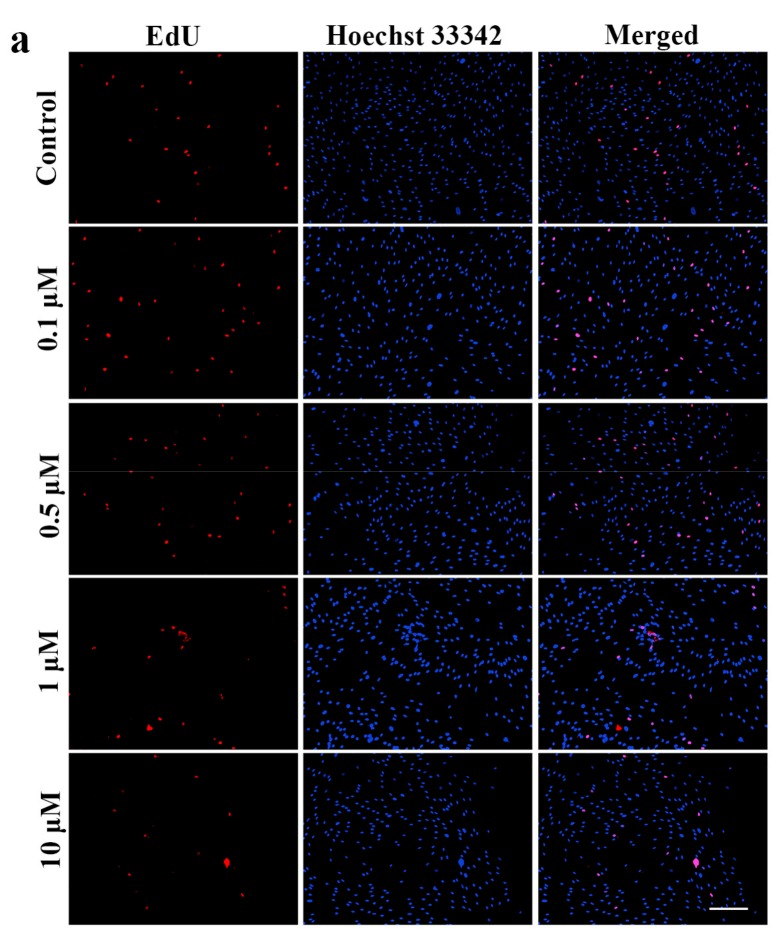

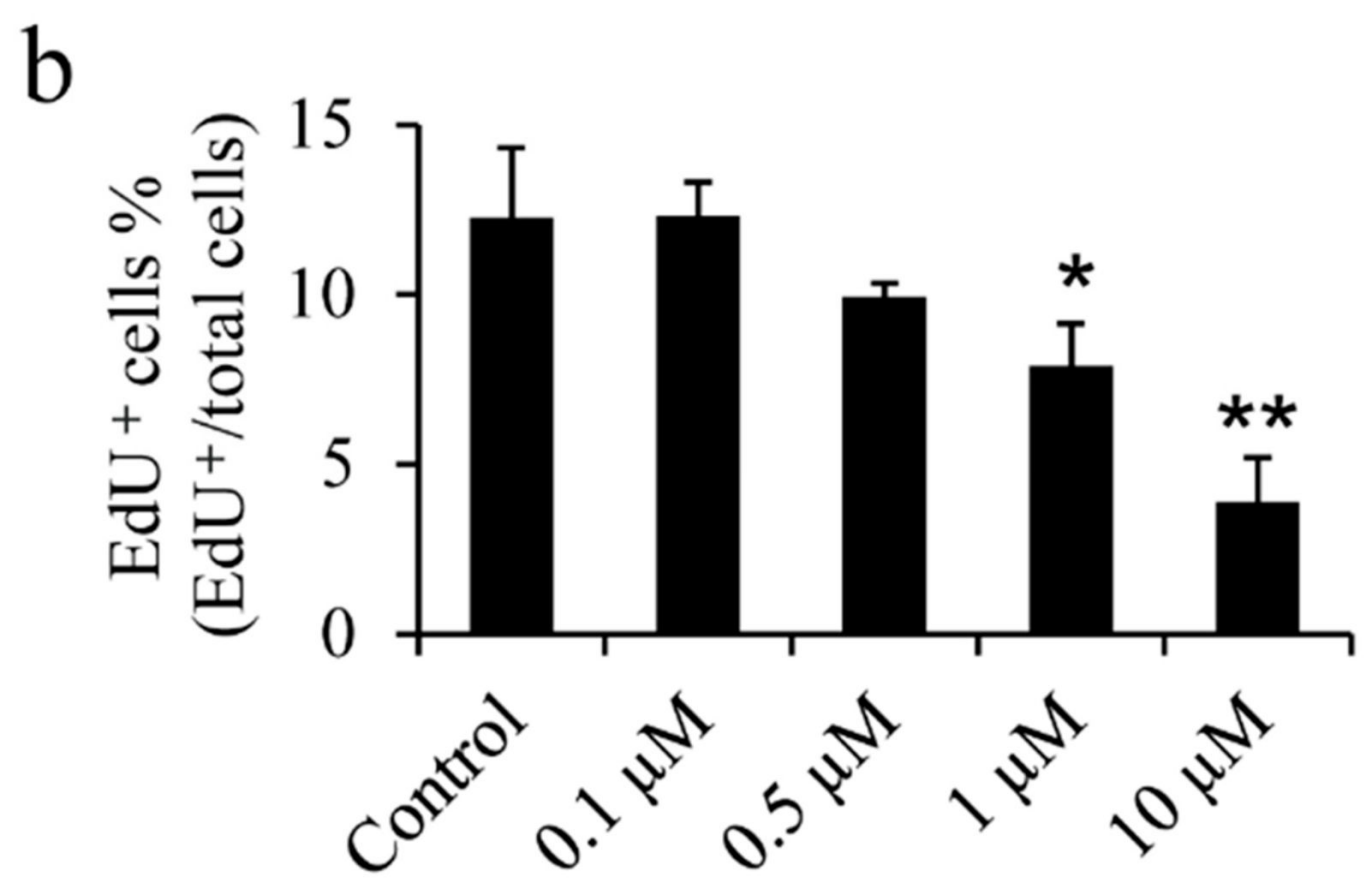
The effect of different concentrations of icariin on cell proliferation of OA–FLSs for 12 h. (**a**) The OA–FLSs were treated with 0.1, 0.5, 1 μM and 10 μM of Icariin. The proliferation of cells was measured by ethynyl deoxyuridine (EdU) assay, EdU staining (red) and Hoechst 33342 staining (blue), scar bar: 200 μm. (**b**) The percentage of EdU^+^ cells based on (**a**) statistical difference when compared to the control (* *p* < 0.05) and significant difference when compared to the control (** *p* < 0.01) (mean ± SD, *n* = 4).

The EdU DNA Proliferation in vitro Detection kit (RiboBio, Guangzhou, China) was adopted to measure the effect of icariin on OA–FLSs proliferation. Amounts of 0.1, 0.5, 1, 10 μM icariin were tested in this experiment to validate the results obtained from the MTS assay. Figure 2a shows the EdU-labeled replicating cells (EdU^+^ cells) (red) and Hoechst 33342-labeled cells (blue) after 12 h treatment of icariin. Compared to the control group, there were less EdU^+^ cells in the field of view after treatment by 1 μM and 10 μM icariin (Figure 2a). When treated with 1 μM icariin, the percentage of EdU^+^ cells was decreased by 0.36-fold (*n* = 4, *p* < 0.05) (Figure 2b). When treated with 10 μM icariin, the percentage of EdU^+^ cells was significantly decreased by 0.68-fold (*n* = 4, *p* < 0.01) (Figure 2b). The results suggest that icariin has a dose-dependent effect on the proliferation of OA–FLSs; 1 and 10 μM concentration of icariin can decrease cell proliferation when compared with the control group.

**Figure 3:**
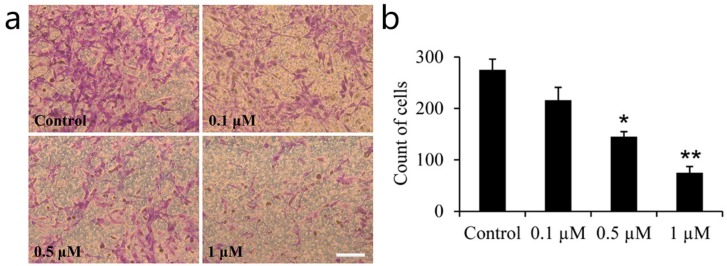
The effect of different concentrations of icariin on cell migration of OA–FLSs for 12 h. (**a**) Cells were treated with 0.1, 0.5 and 1 μM of Icariin.The migrated cellst were stained with crystal violet. (**b**) Statistical analysis of the cell count for the migrated cells. Statistical difference when compared to the control (* *p* < 0.05), significant difference when compared to the control (** *p* < 0.01) (mean ± SD, *n* = 5). Scale bar: 100 μm.

**Figure 4:**
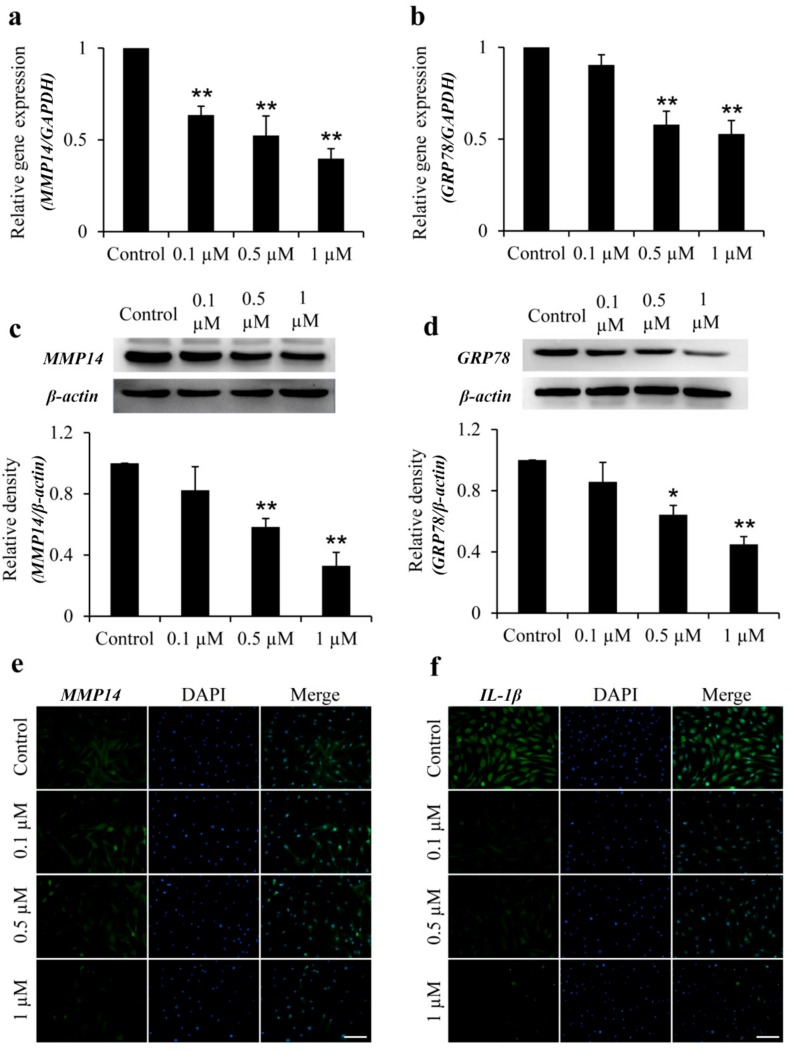
Effects of icariin on *MMP14*, *GRP78*, and *IL-1β* expression in OA–FLSs. (**a**,**b**): The mRNA expression level of *MMP14* (**a**) and *GRP78* (**b**) of OA–FLSs with different concentrations of icariin treatment for 12 h. (**c**,**d**): The protein synthesis of *MMP14* (**c**) and *GRP78* (**d**) of OA–FLSs with different concentrations of icariin treatment for 12 h. Statistical difference when compared to the control (* *p* < 0.05), significant difference when compared to the control (** *p* < 0.01) (mean ± SD, *n* = 3). (**e**,**f**) Immunofluorescence of the *MMP14* (**e**) and *IL-1β* (**f**) of OA–FLSs with different concentrations of icariin treatment for 12 h. Scale bar: 100 μm.

The transwell assay was adopted to detect whether icariin can affect the migration of FLSs (Figure 3). Compared to the control group, the 0.5 μM icariin group experienced a 0.47-fold decrease in OA–FLSs migration, and the 1 μM icariin group experienced a 0.73-fold decrease in OA–FLSs migration (Figure 4b). The result suggests that icariin can inhibit the migration of OA–FLSs.

### 2.2. Icariin Inhibits MMP14 and GRP78 Gene and Protein Expression in OA–FLSs

The expression of *MMP14* and *GRP78* in OA–FLSs treatment with icariin was assessed by using quantitative real-time PCR (qRT-PCR) and western blotting (WB). The icariin could significantly inhibit the gene expression of *MMP14* and *GRP78* in concentration-dependent doses, and the inhibitory effect reached a maximum at 1 μM for *MMP14* and GRP78. Compared with the Control, 1 μM icariin decreased *MMP14* and *GRP78* mRNA levels by 2.6-fold (Figure 4a) and 1.9-fold (Figure 4b) respectively in OA–FLSs. With a similar variation tendency in the protein expression of *MMP14* and *GRP78* in OA–FLSs, the level of *MMP14* and *GRP78* was decreased by 3.4-fold (Figure 4c) and 2.3-fold (Figure 4d) respectively at 1 μM concentration of icariin. Taken together, the higher the concentration of the icariin, the stronger the effect of the inhibition on *MMP14* and *GRP78*. The result suggests that icariin can reduce *MMP14* and *GRP78* expressions in OA–FLSs.

### 2.3. Icariin Inhibits IL-1β and MMP14 Expression in OA–FLSs

Immunofluorescence detected the expression of *IL-1β* and *MMP14* in OA–FLSs treated with icariin (Figure 4e,f). The results show that, compared to the control group, the *IL-1β* and *MMP14* expression was decreased in the icariin group. Additionally, the 1 μM group can significantly inhibit the expression of *IL-1β* and *MMP14*. The result suggests that icariin can reduce *IL-1β* and *MMP14* expression in OA–FLSs.

## 3. Discussion

There is no study about the influence of icariin on FLSs in OA, especially in human cells. Thus, we chose 0.1 to 10 μM of icariin via MTS to detect the cytotoxicity and viability of OA–FLSs. Icariin showed low cytotoxicity to OA–FLSs at a concentration of under 10 μM and decreased the proliferation of the cells at a concentration of 1 and 10 μM. This indicates that 0.1 to 10 μM concentrations of icariin can be used for the proliferation study. Next, we used EdU assay with concentrations from 0.1 to 10 μM to detect the influence of icariin on the proliferation of OA–FLSs. The result showed that icariin has a dose-dependent effect on the proliferation of OA–FLSs; it can forcefully decrease the proliferation of the cells at an icariin concentration of 1 μM and 10 μM compared with the control group. The MTS and EdU assays indicate that 0.1 to 1 μM concentrations of icariin can be used for further study. To gain further insight into the regulation of OA–FLSs behavior by icariin, migration assays were performed. Our results demonstrated that icariin could distinctly inhibit the migration of OA–FLSs and the inhibiting effect has a dose-depended manner.

OA is a complex degenerative joint disease and it develops with a process of mechanical problems, but the inflammation of synovium is also a key event in the pathophysiology of OA [6,7]. *IL-1β* participate in OA synovium inflammation [27], and *IL-1β* is one of the most prominent mediators of cartilage degradation and joint inflammation [28,29]. Our results found that icariin significantly reduced the expression of the pro-inflammation cytokine of *IL-1β*. *IL-1β* signaling suppresses the synthesis of aggrecan and collage [30,31]. In the progress of OA, however, *IL-1β* can stimulate its own producer in an autocrine manner because FLSs contain a high concentration of the *IL-1β* receptor [32]. Therefore, the decrease of *IL-1β* in OA–FLSs proves that icariin has the potential to be a compound for the treatment of OA disease. MMPs are the proteolytic enzymes that contribute to the degradation of ECM [33]. In recent years, studies have shown that *MMP14* has effects on the cartilage tissue in OA disease by degrading the collage II directly and activating other members of MMPs such as *MMP13* [18]. Icariin could attenuate lipopolysaccharide-induced inflammatory responses and reduce ECM degradation through the inhibition of nitric oxide, *MMP1*, *MMP3* and *MMP13* synthesis in murine chondrocytes [26]. Another study proved that Icariin promotes the synthesis of ECM by increasing the expression of aggrecan, *collagen II* and *Sox9* genes in rabbit chondrocytes [34]. Our data showed that icariin could reduce the expression of *MMP14* both at the gene and protein level of FLSs, which further demonstrated the former studies. What is more, the decreasing expression of *MMP14* could reduce the ECM degradation, thus inhibiting the migration of the cells; this confirmed the result of our former experiment about the migration effect of icariin on FLSs.

*GRP78* could aggregate in the ER and activate downstream effectors by binding to unfolded proteins; *GRP78* could also restore the processing capacity of ER protein, as well as restoring the balance of redox and homeostasis of calcium [35,36]. It has been reported that the expression of *GRP78* promotes the apoptosis as well as decreases the synthesis of type II collagen in the chondrocytes of patients with OA [37]. Our results showed that icariin could decrease the expression of *GRP78* of FLSs in vitro, indicating that it regulates OA–FLSs by reducing ER-stress and decreasing the apoptosis of the cells. 

In conclusion, this study demonstrated that icariin at concentrations ranging from 0.1 to 1 μM possessed no cytotoxicity and has an inhibiting effect on the proliferation of FLSs while inhibiting the migration of cells. Icariin could down-regulate the expression levels of *IL-1β*, *MMP14* and *GRP78*. Admittedly, the molecular mechanisms of icariin in the three pathogenic cytokines need to be further investigated. The preliminary results prove that icariin might have a therapeutic effect in the treatment of OA.

## 4. Materials and Methods

### 4.1. Cell Culture

The human materials used for this study were obtained according to ethical principles and the protocol was reviewed and approved (SYXK-CQU-20130021, 21 October 2014) by our Institutional Review Board (IRB).

Human synovial fibroblasts of osteoarthritis (OA–FLSs) were harvested from four donor tissues (aged between 30 and 60) with osteoarthritis undergoing limb amputation at the First Affiliated Hospital of Chongqing Medical University, Chongqing, China. The standard operating procedure of synovial fibroblasts culturing was described in an earlier study [38]. Cells of the passage between 3 and 5 were used in our experiments.

### 4.2. Cytotoxicity Assay

The synovium fibroblasts were trypsinized and seeded onto 96-well plates (Corning, New York, NY, USA) at a concentration of 5 × 10^3^ cells per plate, and cultured in 10% fetal bovine serum (FBS) high glucose Dulbecco modified Eagle medium (DMEM) (0.2 mL per plates). The cells were allowed to seed for 24 h. Then, the culture media were removed and replaced by 2% FBS DMEM during 16 h for starvation (2% FBS high glucose DMEM). Then, the concentrations of 0, 0.1, 0.5, 1, 5 and 10 μM icariin (Ica, purity ≥ 98%, purchased from the company of Solarbio, Beijing, China), which were dissolved in dimethylsulfoxide (DMSO) were chosen for cytotoxicity assay by MTS.

### 4.3. EdU Assay

Based on the results of the MTS assay, we investigated the proliferation of OA–FLSs with different concentrations of icariin treatment by using the EdU DNA Proliferation in vitro Detection kit (RiboBio, Guangzhou, China). The 5 × 10^3^ cells were seeded in a 96-well plate and allowed to culture for 12 h, and then treated with different concentrations of icariin for 12 h. Then, the cells were incubated with EdU-labeling medium (50 μM) for 2 h. The cells were fixed in 4% (*v*/*v*) paraformaldehyde for 30 min and neutralized with glycine in 2 mg/mL. About 5 min later, cells were stained with Apollo fluorescent dye (RiboBio) for 30 min, then 0.25% Triton X-100 was added to each well to permeabilize the stain cells. Finally, cells were stained with Hoechst 33342 and then washed with PBS 3 times. The images of the cells were captured with a fluorescent microscope by Image-Pro Plus 6.0 software (Media Cybernetics, Rockville, MD, USA). The EdU-positive cells from five fields were randomly selected from each well. This experiment was repeated three times.

### 4.4. Transwell Assay

A cell migration assay was performed with a 24-well Milli-cell transwell system (Millipore, Burlington, MA, USA). OA–FLSs were trypsinized and resuspended; 1 × 10^4^ cells were seeded with 2% FBS high glucose DMEM in the upper chamber and 2% FBS-DMEM containing 0.1, 0.5, 1 μM of icariin in the lower chamber. After seeding for 12 h, fixing the samples in 4% paraformaldehyde (PFA) and staining them for 15 min with diamidino phenylindole (DAPI) (Roche, Basel, Switzerland), they were photographed with a fluorescence microscope (Olympus, Tokyo, Japan). Then, five random fields were selected to count the amount of cells for the quantitative analysis. This experiment was repeated four times.

### 4.5. Immunofluorescence Assay

*MMP14* and *IL-1β* were examined by immunofluorescence. After, OA–FLSs were seeded in a 48-well plate and treated with different concentrations of icariin. The cells were fixed in 4% PFA for 30 min; then, 1% Bovine Serum Albumin (BSA) was used to block for 1 h at room temperature, and then incubated with rabbit anti-mouse *MMP14* and *IL-1β* antibody (1:1000, Abcam, Cambridge, UK) at 4 °C overnight. The cells were washed with PBS 3 times, then added to the second antibody which conjuncted with fluorescein isothiocyanate (FITC) and goat anti-mouse IgG antibody (1:100, Abclonal, Woburn, MA, USA); reactions were incubated at 37 °C for 1 h in the dark. Lastly, the cells were counterstained with DAPI (Roche), and the results were observed with the fluorescence microscope (Olympus). The number of cells in five random fields of each well were counted for a quantitative analysis. This experiment was repeated four times.

### 4.6. Quantitative Real-Time Polymerase Chain Reaction

Quantitative real-time PCR was performed to compare the levels of steady-state mRNA for *MMP14* and *GRP78* genes in OA–FLSs treated with different concentrations of icariin. The basic local alignment search tool (BLAST-2.2.9-ia32-win32.exe, NCBI, National Institutes of Health, Bethesda, MD, USA) was used to search for all the primer sequences to ensure gene specificity. Selected sets of primers purchased from Sangon Biotech (Sangon, Shanghai, China) are shown in Table 1. In brief, qRT-PCR was performed with the SsoAdvanced SYBR Green PCR supermix (1725264, Bio-Rad, ‎Hercules, CA, USA) using iCycler (Bio-Rad) according to previously described techniques [39]. PCR reactions were performed in a 10 μL volume with 1 μL cDNA sample and 0.5 μL of each primer. Then, the reaction was initiated by activating the polymerase with a 5 min pre-incubation at 95 °C. Amplification was performed for 40 cycles of 15 s denaturation at 95 °C, annealing at 60 °C for 1 min and extension at 72 °C for 10 s. This experiment was repeated three times. Glyceraldehyde-3-phosphate dehydrogenase (*GAPDH*) was selected as the internal control for normalization.

### 4.7. Western Blotting

The concentration of each sample protein was measured by the bicinchoninic acid (BCA) protein assay kit (Bioteke, Beijing, China). Equal amounts of total protein (50 μg) from each sample were separated in a 10% sodium dodecyl sulfate polyacrylamide gel electrophoresis (SDS-PAGE) gel for 3 h at room temperature and transferred to a polyvinylidene fluoride (PVDF) membrane at 110 V for 2 h on ice. The blot was blocked with 5% nonfat dry milk suspended in 1× Tris buffered saline with Tween (TBST) (25 mM Tris, 2.7 mM KCl, and 137 mM NaCl, 0.05% Tween-20) for 40 min in the incubator at 37 °C. Then, the blot was incubated with ER stress antibody (1:1000, Cell Signaling Technology, Danvers, MA, USA) and MMP14 (1:1000, Abcam) at 4 °C for 12 h, followed by incubation with goat anti-rabbit IgG-HRP (1:10,000, Lianke, Hangzhou, China) for 1 h at room temperature. The blot was washed three times with 1× TBST for 10 min between the first and second incubation. A densitometer (Bio-Rad) to scan the bands and a Quantity One 4.6.3 software (Bio-Rad) was used for quantification. This experiment was repeated four times. 

### 4.8. Statistical Analysis

All experiments were repeated at least three times. The data were presented as the mean ± SD. The one-way analysis of variance (ANOVA) followed by a Tukey test were used to test whether the p values indicated a significant difference between the groups; * p < 0.05 and ** p < 0.01 were deemed to be statistically significant.

## Figures and Tables

**Table 1:** Primer sequences for real-time RT-PCR.

Genes	Primer Sequences (Forward/Reverse)	Product Size (bp)
*MMP14*	5′-CCGATGTGGTGTTCCAGACA-3′	153
	5′-CGTATGTGGCATACTCGCC-3′	
*GRP78*	5′-CTTAAGCTGCCACCATGAAG	256
	CTCTCCCTGGTGGCCGCG-3′	
	5′-AGGCCTCGAGCTACAACT	
	CATCTTTTTCTGCTGT-3	
*GAPDH*	5′-AAATTCCATGGCACCGTCAAGGCT-3′	183
	5′-CTCATGGTTCACACCCATGACGAA-3′	
